# In the Twilight

**Published:** 1876-03

**Authors:** 


### IN THE TWILIGHT.

In the shadow of the twilight, trees are waving to and fro
In a rhythmic measure, keeping time to thoughts of long ago.

Thoughts that swell like far-off music, thrilling me with subtle pain,
Thoughts that nature in her musing, echoes in a sad refrain.

O belovéd! do thy heart-strings quiver in this dreamy eve?
Cans't thou not, in all thy splendor, for the past one moment grieve?

Hast thou grown than nature colder, and forgotten happy days,
When with faith and hope we could not see the parting of our ways?

Moist with dew, this vine its clustering arch of tangled fragrance rears;
Speaks it not of love and spring-time, sweeter through a mist of tears?

And these mosses gray and clinging, fragments caught from twilight's vail,
Tell they not of sorrows, twining all our life with memories pale?

Nay, forgive, forgive this doubting, in thine over-brimming eyes,
Still, I see that worldly living hath not made thee worldly wise.

Was it right or was it sinful thus to let our different spheres
Hold us severed, bound in misery, through such weary length of years?

For thy soul, its fullest stature could not reach except through mine,
And my life so poor, defrauded, could not know its power divine.

Let it pass—O my belovéd!—see, I stifle all my pride;
'Tis not yet too late to finish our short journey side by side.

There the dark-robed clouds are sweeping to the quiet sunlit west;
Shall not thus our past all vanish in Eternity of Rest?

Here where we in childhood wandered, finding flowers for every mood,
I behold one pure and saintly, starlike lighting all the wood.

And I pluck it, for no longer care and pain with us can dwell,
Love shall crown us living, dying, with its fadeless immortelle.

